# Evaluation of BR1 and BI30 AAVs for Brain Endothelial Tropism

**DOI:** 10.1080/17590914.2024.2427953

**Published:** 2024-12-02

**Authors:** Felecia M. Marottoli, Deebika Balu, Rohan Chaudhary, Sarah E. Lutz, Leon M. Tai

**Affiliations:** Department of Anatomy and Cell Biology, University of Illinois at Chicago, Chicago, IL, USA

**Keywords:** AAV, brain endothelial cells, BI30, BR1

## Abstract

Brain endothelial cells are critical for homeostasis of the central nervous system. Novel adeno-associated viruses (AAV) with brain endothelial cell tropism have been developed and are beginning to be employed in mechanistic and therapeutic research. Studies using AAVs can be involved in terms of cost, time and personnel, and many groups, including our own, are not experts on the technology. Therefore, it is important to report data using AAVs with the research community as a guide for ongoing and future studies. Here, we detail our initial experience with the two most prevalent AAVs with tropism for brain endothelial cells, AAV-BR1 and AAV-BI30. One of our long-term goals is to express key proteins in brain endothelial cells and determine the impact on brain function. For method development, we administered AAV-BR1 and AAV-BI30 with a CMV-driven fluorescent reporter (CMV-P2A-mCherry) to wild-type mice intravenously (retro-orbital) and measured expression in brain and peripheral tissues by RT-PCR and immunostaining. We found that AAV-BR1 transduces neurons and endothelial cells in the brain, and the lung and liver, whereas AAV-BI30 transduces brain endothelial cells and peripheral tissue. Our data highlights the importance of using the AAV best suited to the scientific question.

## SUMMARY STATEMENT

Novel AAVs with brain endothelial cell tropism have been developed and are starting to be utilized by researchers for mechanistic and therapeutic studies. Therefore, it is important to report experience with the research community as a guide for future studies and we focused on the two most common AAVs with tropism for endothelial cells, AAV-BR1 and AAV-BI30. We found that AAV-BR1 transduces neurons and brain endothelial cells, and the lung and liver, whereas AAV-BI30 transduces brain endothelial cells, lung and liver. Collectively, our data highlight the importance of using the AAV that best matches the scientific question.

## Introduction

Brain endothelial cells are the central component of the blood-brain barrier and are critical for homeostasis of the central nervous system. Therefore, an important research topic is identifying pathways that regulate brain endothelial cell function in physiological and disease states. To aid in that endeavor, genetic tools are now being developed to modulate brain endothelial cell function. One of the approaches is to modify the capsid of adeno-associated viruses (AAV) to increase brain endothelial cell tropism (reviewed in Lopez-Gordo et al. 2024). Currently, AAV-BR1 (Körbelin et al., 2016) and AAV-BI30 (Krolak et al., 2022) are the most advanced. AAV-BR1 was isolated from an AAV2 library and contains the amino acid sequence “NRGTEWD” in the capsid (Körbelin et al., 2016). AAV-BI30 was identified using a similar approach using AAV9, and contains “NNSTRGG” in the capsid as well as an miR122 transgene in the vector to suppress liver transduction (Krolak et al., 2022). AAV-BR1 and AAV-BI130 are commercially available and are important tools for researchers focused on brain endothelial cell function. One of our long-term goals is to express proteins of interest in brain endothelial cells and determine the impact on brain function. For training and method development, we started to utilize AAV-BR1 and AAV-BI30 with a fluorescent reporter. Over the last year we have found caveats in the use of each AAV regarding cell type and tissue specificity, some of which have been reported by other groups **(**Supplementary File 1, Tables S1 and S2). In this short contribution, our goal is to share our experience with commercially available versions of each AAV and to highlight considerations and suggestions for their use by groups planning experiments similar to our own.

## Methods

The authors declare that all supporting data are available within the article and its online supplementary files.

### Mouse Model and General Design

Experiments were approved by the Institutional Animal Care and Use Committee at the University of Illinois at Chicago. Commercially available C57BL/6J female mice (Jackson Laboratory 000664) were obtained for this study. Mice were housed on a 12 h light/dark cycle with food (Teklad 7912, Inotiv) and water *ad libitum* (see Supplementary File 1 for ARRIVE guidelines).

AAV-(BR1)-CMV-P2A-mCherry and AAV-(BI30)-CMV-P2A-mCherry-3xmiR122 (SignaGen) were diluted in PBS and administered intravenously via injection into the retro-orbital sinus (50 µl) in 6-week-old female mice at 2 x 10^10^ viral genomes (vg), 5 x 10^10^, or 2 x 10^11^ vg/animal. We included a PA2 site, as in future studies our group, and others, may include our protein of interest prior to the cleavage site. Tissues were harvested for analysis about 10 weeks after AAV administration.

### Tissue Harvest

Mice were anesthetized with ketamine (100 mg/kg)/xylazine (10 mg/kg) and transcardially perfused with Tris-buffered saline (TBS). The right hemi-cortex, left lung, and medial lobe of the liver were flash frozen in liquid nitrogen and stored at -80 °C until mRNA extraction for analysis by RT-PCR. For immunohistochemical (IHC) analysis, the left hemi-brain, left lung and remainder of the liver were drop fixed in 3% paraformaldehyde (PFA)/TBS at 4 °C overnight and then transferred to TBS until processing.

### RT-PCR

RNA was extracted using the RNeasy^®^ Plus Mini Kit (Qiagen). Briefly, <20 mg of tissue was homogenized using a bead mill homogenizer (6 m/s, 30 s, 1 cycle), RNA was extracted according to the manufacturer protocol, flash-frozen in liquid nitrogen and stored at −80 °C. RT-PCR was performed by the Genomics Research Core at the UIC. RNA samples were quantified and evaluated for quality using RNA ScreenTape Assay and Agilent 4200 TapeStation. Expression profiling was performed using the 96.96 Gene Expression IFC and data was collected using BioMark HD Real-Time PCR system (Standard BioTools). RNA samples were processed according to Real-Time-PCR-Analysis_68000088-N1 protocol. TaqMan gene expression assays for *Actb* and *mCherry* were ordered from Life Technologies (Applied Biosystems^™^, Supplementary File 1, Major Resources Table). In brief, RNA in the amount of 10–90 ng per sample was converted to cDNA and followed by Target Specific Amplification pre-amplification performed with a master mix of all intended assays. All samples were pre-amplified with 18 PCR cycles. Final cDNA products were diluted 1:5 and used for setting up RT-PCR reactions with each individual assay. Each sample was tested in 6 technical replicates. Fluidigm Real-Time PCR Analysis software was used to collect the data with the following analysis parameters: Quality Threshold at 0.5, Baseline Correction Linear, and Ct Threshold Method on Auto.

### Immunohistochemical Staining

Drop-fixed tissues were cryoprotected in 30% sucrose and sectioned on a sliding microtome for IHC staining. Hemi brains were sectioned along the sagittal plane at 35 µm and stained as free-floating sections. Lung and liver were sectioned at 20 µm and 14 µm, respectively, and mounted on slides prior to IHC. For IHC, sections were washed (6 x 5 min in TBS), permeabilized with dilution media (DM, 0.1% Triton X-100 in TBS, 3 × 10 min) and background PFA quenched (0.05 M ammonium chloride, 10 min). Sections were then rinsed (3 x TBS), blocked (4% BSA in DM, 1 h), and incubated with primary antibodies (1:250) against CD31 (AF3628, R&D Systems) or NeuN (MAB377, EMD Millipore) and red fluorescent protein (RFP) (5F8, Proteintech) overnight (4 °C, humidified chamber, 1% BSA in DM). Following incubation with primary antibody, sections were washed (3 x 5 min in DM followed by 3 x 5 min in TBS), incubated with Alexa Fluor-conjugated secondary antibodies (2 h, room temperature), washed (6 x 5 min in TBS), dried for 30–45 min and cover slipped. Slides were imaged at 10X magnification on an ImageXpress Micro (Molecular Devices) under identical exposure settings. High resolution confocal images were acquired on an LSM 900 (ZEISS) as Z-stacks at 63X magnification under identical exposure settings and converted into maximum projection images using Zen Blue (ZEISS) software. In Supplementary File 1, Figure S1, (AAV-BR1) mCherry fluorescence was imaged in the 594 nm channel. For all other images were immunostained with an RFP antibody and imaged in the 647 nm channel.

### Cortical AAV Tropism Quantification

To quantify the tropism of each AAV, a script was developed for use in ImageJ (Fiji) software to measure the percentage of vessels that were positive for mCherry (via RFP) and the proportion of RFP signal that was due to vessel expression. Briefly, the whole cortex was traced (Serial sagittal sections,three sections between ∼0.72-mm and 1.80-mm lateral), images were processed to remove noise and binarized. The binarized images were used to create ROIs comprised of each individual signal area for each wavelength. The ROIs were then applied to binarized masks to determine the percentage of RFP+ vessels and vascular RFP. The corresponding script can be found in Supplementary File 2.

### Image Processing

ImageJ 1.53c (Fiji), Adobe Photoshop 25.9.1, and Adobe Illustrator 28.5 were used to process images displayed in figures throughout the manuscript. In cases where AAVs were directly compared to vehicle controls, matched images were processed identically.

### Statistical Analysis

All data are expressed as either the mean ± SEM or as a box plot depicting the minimum score, the lower quartile (25%), the median (50%, horizontal line), the upper quartile (75%), maximum values, and mean (+). Data were analyzed with general linear models in SPSS (v28.0.1.1; 15) with Bonferroni post hoc as required.

### Supplementary Files

Supplementary File 1 contains literature summary tables for BR1 and BI130 (Tables S1–S2), Figures (Figures S1–S4), source data and statistical analysis, a Major Resource Table and ARRIVE guidelines. Supplementary File 2 is the Fiji script for quantification of for vascular transduction efficiency (% of RFP+ vessels, % vessel vs non-vessel staining).

**Figure 1.**
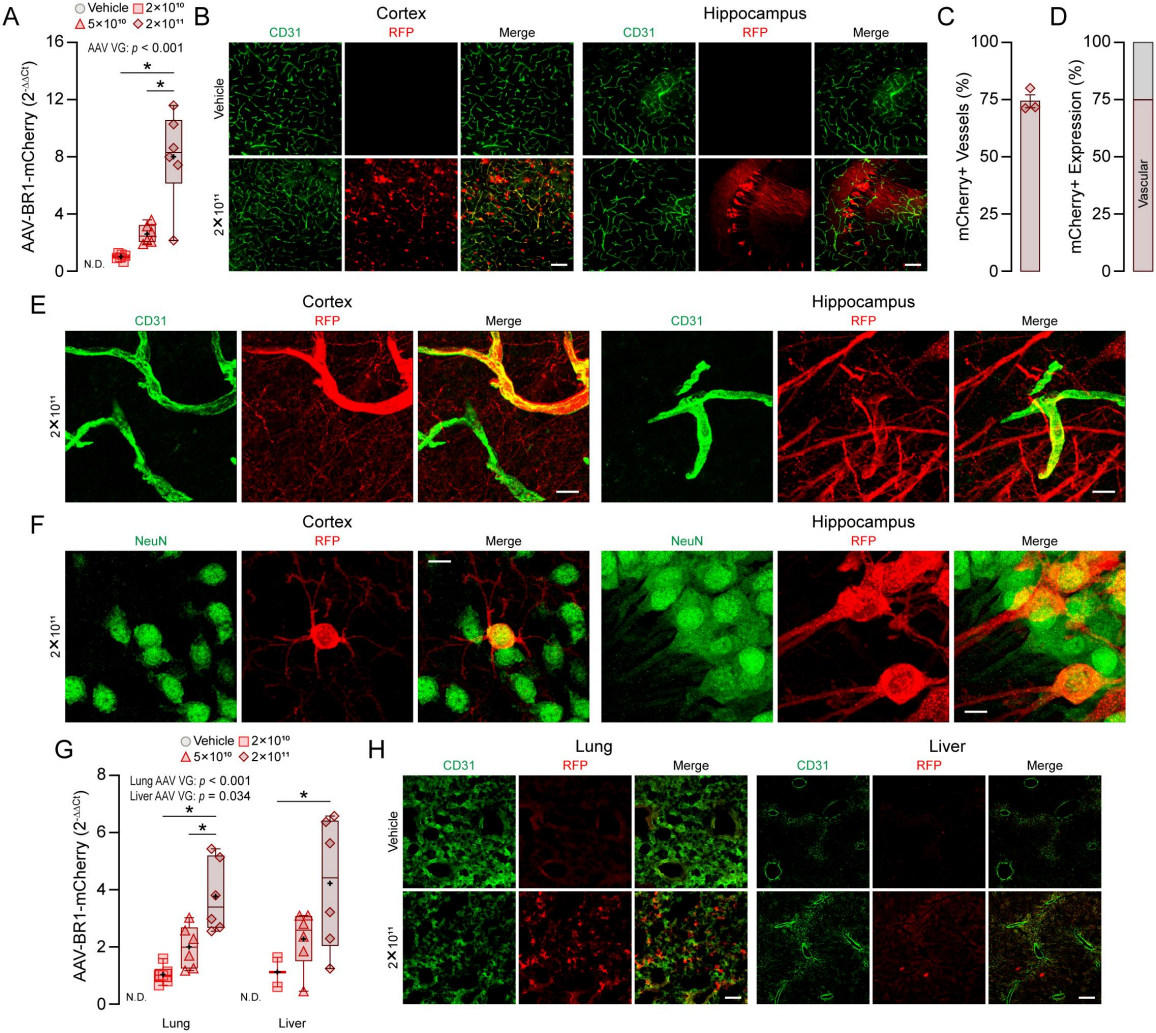
AAV-BR1 shows tropism for brain endothelial cells and neurons. AAV-BR1 containing CMV-P2A-mCherry was administered intravenously (retro-orbital) to 6-week-old female C57BL/6J mice and analysis conducted ∼ 10 weeks later. (A) AAV-BR1-mCherry expression levels in the cortex measured by RT-PCR. AAV-BR1-mCherry expression as fold-change (2^-ΔΔCt^ normalized to 2 x 10^10^ vg) is higher with increasing AAV-BR1-mCherry vg in a dose-dependent manner in the cortex [*F*_(2,17)_ = 21.852; *p* < 0.001], *n* = 6. AAV-BR1-mCherry is not detected in vehicle-treated mice in the cortex. (B) After antibody staining, RFP (mCherry) fluorescence is detected in both endothelial cells and neurons in the cortex (left) and hippocampus (right). Sections were counterstained for CD31. Scale bar = 100 µm. (C–D) Tissue sections immunohistochemically labeled for CD31 and RFP were quantified to determine the endothelial specificity of AAV-BR1-mCherry (2 x 10^11^ vg) in the cortex. (C) ≈ 74.35% of vessels are positive for RFP, *n* = 3. (D) Vascular RFP signal comprises ≈ 74.7% of total AAV-BR1-mCherry transfection, n = 3. (E–F) Maximum projection images of tissue sections immunohistochemically labeled for CD31 or NeuN and RFP taken at 63X magnification were acquired to confirm cell type expression of AAV-BR1-mCherry (2 x 10^11^ vg) in the cortex and hippocampus. Scale bar = 10 µm. (E) High-resolution images revealed substantial non-endothelial RFP (mCherry) fluorescence in both the cortex (left) and hippocampus (right). (F) RFP (mCherry) co-labeled with NeuN revealed RFP (mCherry) fluorescence in the cell bodies and processes of neurons in both the cortex (left) and hippocampus (right). (G) AAV-BR1-mCherry expression levels in the lung and liver measured by RT-PCR. AAV-BR1-mCherry expression as fold-change (2^−ΔΔCt^ normalized to 2 x 10^10^ vg) is higher with increasing AAV-BR1-mCherry vg in a dose-dependent manner in the lung [*F*_(2,17)_ = 15.414; *p* < 0.001], *n* = 6, and in the liver [*F*_(2,14)_ = 4.445; *p* = 0.034], *n* ≈ 6. AAV-BR1-mCherry is not detected in vehicle-treated mice in the lung or liver. (h) RFP fluorescence is detected in both lung (left) and liver (right). Sections were counterstained for CD31. Scale bar = 100 µm. Data in (A) and (G) are expressed as a box plot depicting the minimum score, the lower quartile (25%), the median (50%, horizontal line), the upper quartile (75%), maximum values, and mean (+). Data were analyzed using GLM. Data in (C) and (D) are expressed as mean ± SEM.

## Results

**Figure 2.**
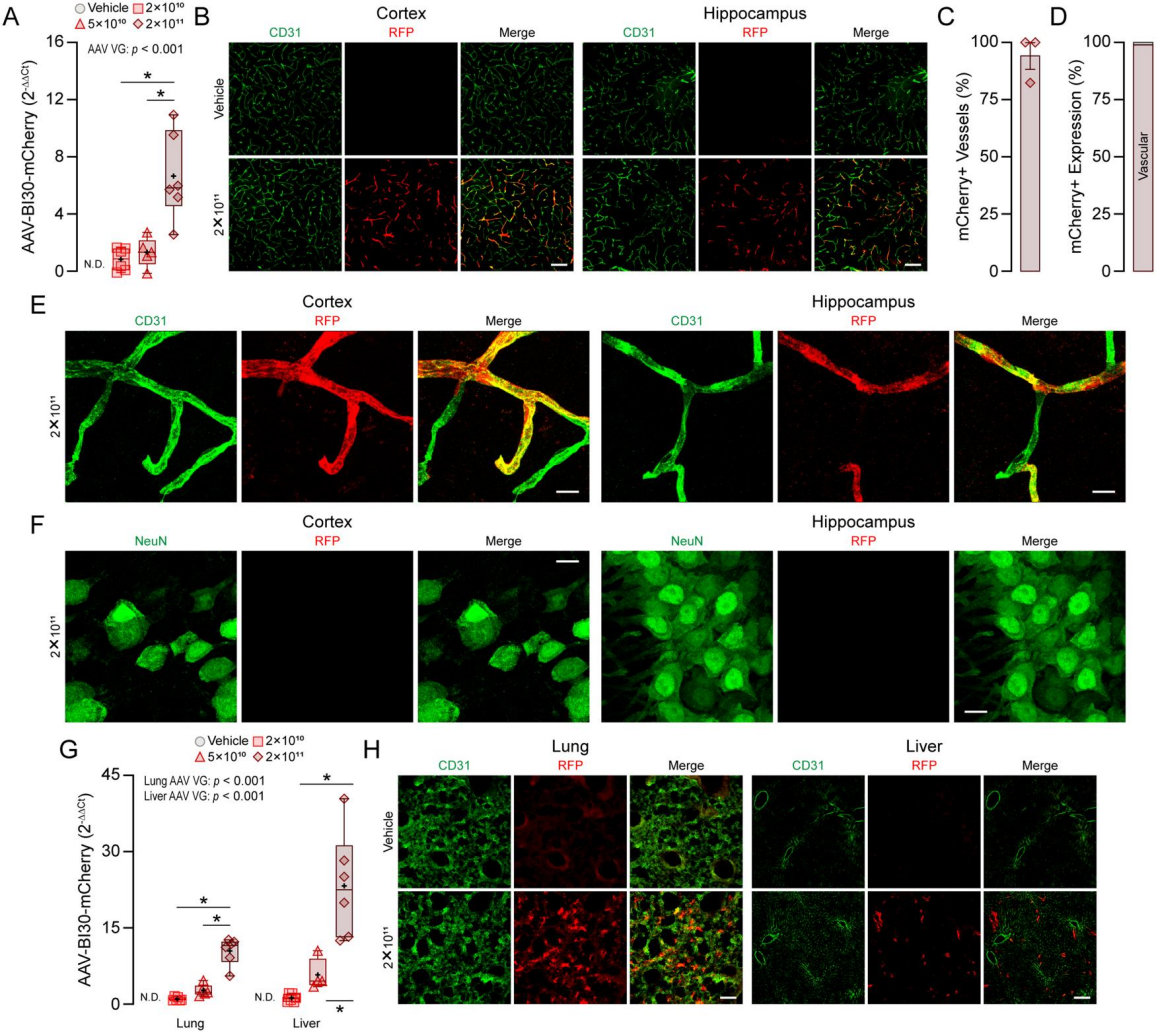
AAV-BI30 demonstrates brain endothelial-specific tropism within the brain. AAV-BI30 containing CMV-P2A-mCherry was administered intravenously (retro-orbital) to 6-week-old female C57BL/6J mice and analysis conducted ∼ 10 weeks later. (A) AAV-BI30-mCherry expression levels in the cortex measured by RT-PCR. AAV-BI30-mCherry expression as fold-change (2^-ΔΔCt^ normalized to 2 x 10^10^ vg) is higher with increasing AAV-BI30-mCherry vg in a dose-dependent manner in the cortex [*F*_(2,16)_ = 9.791; *p* = 0.002], *n* ≈ 6. AAV-BI30-mCherry is not detected in vehicle-treated mice in the cortex. (B) After antibody staining, RFP (mCherry) fluorescence is detected in endothelial cells in the cortex (left) and hippocampus (right). Sections were counterstained for CD31. Scale bar = 100 µm. (C–D) Tissue sections immunohistochemically labeled for CD31 and RFP were quantified to determine the endothelial specificity of AAV-BI30-mCherry (2 x 10^11^ vg) in the cortex. (C) ≈ 94.14% of vessels are positive for RFP, *n* = 3. (D) Vascular RFP signal comprises ≈ 98.75% of total AAV-BI30-mCherry transfection, n = 3. (E–F) Maximum projection images of tissue sections immunohistochemically labeled for CD31 or NeuN and RFP taken at 63X magnification were acquired to confirm endothelial specificity of AAV-BI30-mCherry (2 x 10^11^ vg) in the cortex and hippocampus. Scale bar = 10 µm. (E) High-resolution images revealed endothelial RFP (mCherry) fluorescence located almost exclusively in the endothelial cells of the neurovasculature in both the cortex (left) and hippocampus (right). (F) RFP (mCherry) was not observed in the cell bodies or processes of neurons in the cortex or hippocampus when co-stained with NeuN. (G) AAV-BI30-mCherry expression levels in the lung and liver measured by RT-PCR. AAV-BR1-mCherry expression as fold-change (2^−ΔΔCt^ normalized to 2 x 10^10^ vg) is higher with increasing AAV-BR1-mCherry vg in a dose-dependent manner in the lung [*F*_(2,16)_ = 13.026; *p* < 0.001], *n* = 6, and in the liver [*F*_(2,16)_ = 24.234; *p* < 0.001], *n* ≈ 6. AAV-BR1-mCherry is not detected in vehicle-treated mice in the lung or liver. (h) RFP fluorescence is detected in both lung (left) and liver (right) tissues. Sections were counterstained for CD31. Scale bar = 100 µm. Data in (A) and (G) are expressed as a box plot depicting the minimum score, the lower quartile (25%), the median (50%, horizontal line), the upper quartile (75%), maximum values, and mean (+). Data were analyzed using GLM. Data in (C) and (D) are expressed as mean ± SEM.

Our goal was to understand the extent that AAV-BR1 and AAV-BI30 selectively transduce brain endothelial cells. Therefore, we administered AAV-BR1 or AAV-BI30 containing CMV-P2A-mCherry intravenously (retro-orbital) to 6-week-old female C57BL/6J mice and assessed transduction ≈ 10 weeks later using RT-PCR and quantitative IHC. We treated mice with 2 x 10^10^ viral genomes (vg), 5 x 10^10^, or 2 x 10^11^ vg/animal. The doses were selected based on the range utilized by other groups (Supplementary File 1, Table S1). As we were following published data, vg amounts were per mouse rather than per g, however the average weight of a 6-week-old female mouse is ∼19 g, and so our doses would correspond to 0.1 x 10^10^, 0.26 x 10^10^ and 0.1 x 10^11^ vg/g. We limited our study to female mice as our goal was to compare tropism between the two capsids in a first trial, however we acknowledge that sex could impact AAV transduction. An important note is that for AAV-BR1, staining is visible without the need for use of an RFP antibody for IHC (Supplementary File 1, Figure S1); however, an antibody is required AAV-BI30. Therefore, for consistency, we utilized an RFP antibody in Figures 1 and 2.

### AAV-BR1 Transduces Both Brain Endothelial Cells and Neurons

AAV-BR1 was one of the first AAVs developed with tropism for brain endothelial cells (Körbelin et al., 2016) and has been used in numerous studies (Alvarez-Vergara et al., 2021; Chen et al., 2021; Chen et al., 2020; Dogbevia et al., 2017, 2020; Huo et al., 2023; Ivanova et al., 2022; Johann et al., 2023; Kremer & Williams, 2024; Kawabata et al., 2023; Körbelin et al., 2016; Li et al., 2023; Liu et al., 2020; Mehina et al., 2021; Nikolakopoulou et al., 2021; Park et al., 2021; Rasmussen et al., 2023; Ren et al., 2021; Sundaram et al., 2022; Song et al., 2022; Santisteban et al., 2020; Toma et al., 2024; Tan et al., 2019; Wang et al., 2019). Therefore, we first evaluated the effect of AAV dose on AAV-BR1 transduction in the brain (Figure 1). We initially measured levels of mCherry in the cortex via RT-PCR and found that transduction levels increase with increasing dose or vg load (lower ΔCt values, Figure 1A, Supplementary File 1, Figure S2A). After confirming cortical transduction, we next evaluated the brain regions and cell types that were transduced by AAV-BR1 (Figure 1B and Supplementary File 1, Figure S1). As expected, there was brain endothelial cell expression throughout the brain. Surprisingly, we found high RFP fluorescence in neurons of various brain regions, including the cortex and CA2 region of the hippocampus (Figure 1B), as well as the globus pallidus, and interposed nucleus of the cerebellum (Supplementary File 1, Figure S1). Quantitatively, for vascular transduction efficiency in the entire cortex, we found that AAV-BR1 transduced ≈ 75.35% of vessels (Figure 1C) and that brain endothelial cells accounted for ≈ 74.7% of mCherry transduction (Figure 1D). We also validated qualitatively that mCherry was expressed in brain endothelial cells and neurons using higher magnification images in the cortex and hippocampus (Figure 1E,F). We next evaluated the extent that AAV-BR1 transduced the brain compared to other organs. We found mCherry in both lung and liver when assessed by RT-PCR (Figure 1G, Supplementary File 1, Figure S2B) and IHC (Figure 1H). Collectively, these data suggest that AAV-BR1 demonstrates tropism for brain endothelial cells, neurons, and peripheral tissues to varying degrees.

### AAV-BI30 is Endothelial Cell-Specific in the Brain

AAV-BI30 is a more recently developed AAV with reports of high transduction efficiency in brain endothelial cell (Giannelli et al., 2024; Krolak et al., 2022), and is beginning to be utilized by several groups. We therefore determined the extent that AAV-BI30 transduced brain and peripheral cells using the same approach as for AAV-BR1. AAV-BI30 transduction increased with vg dose when assessed by RT-PCR (Figure 2A, Supplementary File 1, Figure S3A). When assessed by IHC, we found RFP signal throughout the brain that appeared exclusive to brain endothelial cells, as neuronal expression appeared absent (Figure 2B and Supplementary File 1, Figure S4). Indeed, when we quantified the vascular transduction efficiency of AAV-BI30, ≈ 94.14% of vessels were RFP+ (Figure 2C) and brain endothelial cells accounted for ≈ 98.75% of RFP transduction (Figure 2D). We also validated qualitatively that mCherry was expressed in brain endothelial cells and not neurons using higher magnification images in the cortex and hippocampus (Figure 2E,F). In the periphery, as observed with AAV-BR1, RT-PCR (Figure 2G, Supplementary File 1, Figure S3B) and IHC staining (Figure 2H) revealed expression in both the lung and liver. Our data is consistent with other reports that AAV-BI30 demonstrates highly specific tropism for endothelial cells within the brain, but also results in expression in lung and liver tissues.

## Discussion

Brain endothelial cells are critical for neuron function and are disrupted in many neurodegenerative disorders. Therefore, an important focus is understanding how brain endothelial cell proteins impact cerebrovascular and brain function. AAVs are powerful tools, and research using AAVs with tropism for brain endothelial cells can range from development of new AAVs to expression or knockdown studies in wild-type or disease-relevant models. The latter application encompasses several groups who, like us, may have experience with brain endothelial cell function but are not experts on AAVs. For many smaller-medium size laboratories the costs in time, personnel and resources limit the extent that reagent testing, and validation can be conducted. This issue applies to all research and is particularly problematic for more complex studies that include aged mice of multiple genotypes. Although new AAVs continue to be developed, understandably the most published and commercially available reagent is often utilized. Thus, reporting experience with AAVs with tropism for brain endothelial cells is important for helping to guide research programs. Here, we detailed our initial experience with AAV-BR1 and AAV-BI30. Our overall intent is in no way to diminish or question their value in research, as they are excellent tools that we will incorporate, but to share our experience with the commercially available versions and offer suggestions. Our data supports that ultimately the use of a particular AAV depends on the research question. If investigators plan to express a protein that only shows activity in brain endothelial cells, a strong advantage of AAV-BR1 is the high levels of expression. We consider BR1 to have high expression because an RFP antibody was not needed to image expression unlike BI30, and levels appear higher than BI30 in the brain when assessed by RT-PCR. In addition, neuronal expression may increase chances of activity through protein secretion that can act on brain endothelial cells. If it is important that a protein is only produced by brain endothelial cells in the brain, then AAV-BI30 is likely more optimal. A potential caveat for both is that in our hands we found transduction in peripheral tissues (lung and liver) for both AAVs. Peripheral transduction does not invalidate the use of the AAVs, as the high level of brain endothelial cell expression is likely to be more proximal to any functional effects in the brain. However, it is important to check and report in case future studies link peripheral effects of a protein to a brain phenotype. If a lack of peripheral transduction is essential for addressing a specific research question, then brain endothelial cell-specific promoters can be used in place of CMV or CAG. That approach will involve testing multiple different promoters for relative expression levels in endothelial cells from different tissues. In addition, it may be important to determine transcriptional activity of the selected protomer in the model(s) of use prior to introducing the AAV as it would impact the expression of the protein encoded in the AAV. Our recommendation is to first test with the fluorescent reporters, decide which AAV is optimal for the research purpose, and then conduct larger studies.

An important step in designing an AAV experiment *in vivo* is evaluating cost/feasibility and we will use our research as an example. Our overall goal is to express two different proteins in brain endothelial cells using multiple mouse models in sequential phases. In the first phase we will transduce mice that lack our proteins of interest in all cell types and compare them to control mice with global production of the proteins. We conducted the study described in this manuscript for method development and are going to select AAV-BI30, as it is essential that we do not have neuronal expression, and we will check/report peripheral expression with a variety of techniques. The total cost of this current study including analysis but excluding personnel was ∼$10,000 and took around 6 months. Reporting results from studies such as ours is important, as they are costly, take time, and can help inform other research groups on the advantages and disadvantages of each AAV. Selecting the right AAV at the start of larger scale projects is critical, as the costs in personnel, time and resources are much higher. As an example, in our second phase the costs of AAVs will be ∼$50,000–$100,000 (at 2 x 10^11^ vg/animal) and the experiments will take ∼1.5–2 years to complete. In our subsequent phases using additional mouse models, for consistency we would prefer to continue to use BI30. However, it is likely that as new AAVs are developed and characterized, and expectations from grants and manuscripts shift, that we will have to repeat the studies described in this manuscript.

We would like to reiterate and acknowledge some limitations in the current manuscript. As described above, we focused on female mice, however sex could influence AAV expression and tropism, and our doses were not based on body weight, which may be important to incorporate in future studies. In terms of peripheral expression, we have thus far not identified the cell types that are transduced by AAVs, and due to a lack of expertise at the time did not inflate the lungs to enable quantification. For quantification of mCherry expression by brain endothelial cells we wanted to evaluate as much tissue as possible, and we therefore imaged at 10x magnification and traced the whole cortex. High power magnifications revealed that AAV-BI30 appears expressed mainly, if not exclusively by brain endothelial cells. However, BR1 is expressed by non-brain endothelial cells, which despite our best quantification intentions could have impacted quantification. For example, expression in other cell types above or below the plain of imaging could have interfered with the quantification result. We also focused on young mice, however altered blood-brain barrier function during aging and in models of disease may impact AAV expression. In general, the blood-brain barrier is considered fully formed and mature from birth, however during aging and in neurodegeneration brain endothelial cell function could be altered, such as higher permeability, changes in mRNA and protein turnover and modified angiogenic signaling, which could impact expression in brain endothelial cells and disruption to the brain. In our study, we found that BI30 was expressed in the liver, which is surprising since it was developed to not target the liver by using miR122 as a de-targeting strategy. We are unsure of the underlying reason, but it could be plausible that miR122 does suppress liver expression compared to its absence but is not a full prevention of expression.

## Supplementary Material

Marottoli_Balu et al AAV_Supp File 2_ASN Neuro_Submission 2.zip

Marottoli_Balu_et_Supp 1.docx

## Abbreviations

AAV: adeno-associated viruses
AAV-BR1: AAV-BR1 AAV2 containing the amino acid sequence “NRGTEWD” in the capsid
AAV-BI30: AAV-BI30 AAV9 containing the amino acid sequence “NNSTRGG” in the capsid
RFP: red fluorescent protein.
