# Supplementary material for: Evaluation of BR1 and BI30 AAVs for Brain Endothelial Tropism
Source: ASN Neuro. 2024 Dec 2;16(1):2427953. doi: 10.1080/17590914.2024.2427953 (PMC11792159; doi:10.1080/17590914.2024.2427953)
Supplement: Marottoli_Balu_et_Supp 1.docx [file TASN_A_2427953_SM8264.docx]

**SUPPLEMENTARY FILE 1**

**Evaluation of BR1 and BI30 AAVs for brain endothelial tropism**

Felecia M. Marottoli^1#^, Deebika Balu^1#^, Rohan Chaudhary^1^, Sarah E Lutz^1^, Leon M. Tai^1*^

**Author affiliations**:

^1^Department of Anatomy and Cell Biology, University of Illinois at Chicago, Chicago, IL, United States.

^#^Equal contributions

*Corresponding author: [leontai@uic.edu](mailto:leontai@uic.edu)

**This file contains:**

- Tables S1. Literature summary for BR1. Pages 2-4.
- Tables S1. Literature summary for BI130. Page 5.
- Figure S1-4. Page 6-9.
- Source Data and statistical analysis. Page 10-15.
- Major resource Table. Page 16.
- ARRIVE guidelines. Page 17.
- References. Pages 18-19.

**Table S1. Summary of published data for AAV-BR1 selectivity in rodents**

| **AAV construct** | **Model, Age at Tx** | **Dose, route of admin** | **AAV duration** | **BEC expression** | **Non-BEC expression (CNS)** | | **Peripheral expression** | | **Ref** |
| --- | --- | --- | --- | --- | --- | --- | --- | --- | --- |
|  |  |  |  |  | **Y/N/NR** | **Cell Type** | **Y/N/NR** | **Organ** |  |
| **CAG** or **CMV**-luciferase | FVB mice, 8-12 wks | 5×10^10^ gp/mouse, tail vein | 14 d | Y (BC) | Y | Neuron (IHC) | Y | Heart, liver*, lung, kidney*, muscle* (BC) | (Korbelin et al., 2016) |
|  |  |  | 8 wks | Y (IHC) | NR | - | Y | Liver*, heart*, kidney*: (IHC) |  |
| **CAG**-EGFP | Ai14 mice, 6 wks | 1.8×10^11^ gp/mouse, tail-vein | 2 wks | Y (IHC) | NR | - | NR | - |  |
| **CAG**-NEMO, control eGFP | Nemo^beKO^ mice, 10-20 wks | 1.8×10^11^ gp/mouse, tail-vein | 60 d | NR | NR | - | N | Liver (?) | (Dogbevia et al., 2017) |
| **CAG**-EGFP | Sema3G^fl/fl^, 8 wks | 3×10^11^ gp/mouse,  intravenous | 3 wk | Y (IHC) | Y | Neurons (IHC) | N | - | (Tan et al., 2019) |
| **CAG**-EGFP  **CAG**-DsRed  **CAG**-DsRed-2A-MCT1  (Dr. Korbelin) | Pten^fl/-^, Pten^fl/fl^, age unknown | 5×10^10^gp/mouse, tail vein | NR | Y (IHC) | NR | - | NR | - | (Wang et al., 2019) |
| **CAG**-EGFP | Cdk5fl;/fl mice, 4 wks | Dose unknown, tail vein | 3 wks | Y (IHC) | NR | - | NR | - | (Liu et al., 2020) |
| **CAG**-iCre-EGFP | Cdh5-Cre; Ai14, 4 wks | Dose unknown, tail vein | 3 wks | Y (IHC) | Y | Neurons (IHC) | NR | - |  |
| **CAG**-iCre-EGFP | Cdh5-CreERT2; Ai14 mice, 4 wks |  | 3 wks | Y (IHC) | NR | - | NR | - |  |
| **CAG**-*HEXA*, **CAG**-*HEXB,* control CAG-Ø | Hexb−/− mice, P0 | 3×10^10^ gp/mouse 50μl, retro-orbital | 14 d | Y (ISH) | NR | - | Y | Liver*, lung* (BC) | (Dogbevia et al., 2020) |
|  | Hexb−/− mice, P30 | 1.8×10^11^ gp/mouse 100μl, retro-orbital | 80-85 d | Y (ISH) | NR | - | Y | Liver*, lung* (BC) |  |
| **CMV**-mScarlet-3FLAG | C57BL/6 mice, 8-16wk | 1.6×10^10^ vg/mouse, icv | 21 d | N | NR | - | NR | - | (Chen et al., 2020) |
| **CAG-**GFP  (Dr. Korbelin) | *Lrp1^lox/lox^; Tie2-Cre* mice, 3M | 2×10^11^gp/mouse 100μl, tail vein | 30 d | Y (IHC) | NR |  | NR | - | (Nikolakopoulou et al., 2021) |
| **CAG**-BR1-iCre  (Dr. Korbelin) | Ai14 ROSA26^TdTomato^ mice (7-10wk) | 1.8×10^10^ vg, 100μl (Route: unknown) | 3 wks | Y (IHC, flow cyto) | NR | - | N |  | (Santisteban et al., 2020) |
| **CAG**-shControl or shPlexinD1 | Ai14 mice, Cdh5-Cre Sema3G^fl/fl^ mice, P17 | 7×10^10^ vg/mouse, retro-orbital | 2d | Y (IHC) | NR | - | NR | - | (Chen et al., 2021) |
| **CAG**-Cre  (Dr. Korbelin) | Ai14 mice, 12 wk | 2×10^9^gp, 200nl, intracerebral | 2 wks | Y (IHC) | Y | Neurons (IHC) | NR | - | (Ren et al., 2021) |
| **CAG**-Cre  (Dr. Korbelin) | Psen1loxP/loxP; Psen2–/– mice, 3 month | 5×10^10^gp/mouse, tail vein | 19 d | Y (ISH) | NR | - | NR | - | (Alvarez-Vergara et al., 2021) |
| AAV-BR1-**CAG**-Cre  (in-house) | Ai9 mice, age unknown | NR | NR | NR | NR | - | NR | - | (Mehina et al., 2021) |
| **CAG**-KRASG12V-WPRE, control eGFP | C57BL/6 mice, 5 wk | 5×10^10^ gc/mouse, 100μl, retro-orbital | 4 wks | Y (IHC, BC) | NR | - | N | Liver (IHC) | (Park et al., 2021) |
| **CMV-**sgCtnnb1-tdTomato  (Dr. Korbelin) | Tie2^Cas9^ mice, P30 | 1.8×10^11^ vg/mouse, tail vein | 4 wks | Y (BC, IHC) | NR | - | N | Lung, liver, kidney (BC) | (Song et al., 2022) |
| **CAG**-Mct8-WPRE , control GFP | Mct8;Oatp1c1 DKO mice, P0 | 10^12^ gc/ml,  100μl, temporal facial vein | 33 d | Y (IHC) | Y | Neurons, Astrocytes (IHC) | N | Kidney, liver (IHC) | (Sundaram et al., 2022) |
|  | Mct8;Oatp1c1 DKO mice, P30 | 4.5×10^12^ gp  75μl, tail vein | 51 d | Y | Y | Neurons, Astrocytes (IHC) | NR | - |  |
| **CAG**-GFP (Virovek CA) | C57BL/6J mice, P60 | 1×10^12^ gc/ml, 100μl tail vein or 1.5μl intravitreal | 1 M | Y (IHC) | Y | Horizontal cells, ganglion cells (IHC) | NR | - | (Ivanova et al., 2022) |
| **CAG**-DIO-Cx43-P2A-DsRed2 (Virovek CA) | Cdh5CreERT2^+/−^ mice, P60 | 10^12^ gc/ml  100μl, tail vein | 4 wks | Y | N | - | NR | - |  |
| **CAG**‐Luciferase | BALB/cJRj mice, 7-8 wks | 5×10^10^ vg/mouse, tail vein | 8 wks | NR | NR | NR | Y | Lung (BC, imaging) | (Rasmussen et al., 2023) |
| **CAG-**NPC2‐eGFP |  | 1.5×10^10^ vg/mouse, tail vein |  | Y (IHC) | Y | Neurons (IHC) | Y | Lung (BC) |  |
| BR1: **CBh**-GFP-WPRE-HBGpA | C57BL/6J mice, adult | 1×10^11^ vg/mouse 100μl, retro-orbital | 3 wks | Y (BC) | N | - | N | Liver (IHC) | (Kawabata et al., 2023) |
| **CAG**-MAP3K3^I441M^-eGFP, control eGFP | C57BL/6 mice, P1 | 3×10^10^gc  10μl, retro-orbital | 6 wks | Y (IHC) | NR | - | N | Liver, kidney (IHC) | (Huo et al., 2023) |
| Cre-**CAG** (Dr. Korbelin) | Krit1^fl/fl^;iPik3ca^H1047R^ mice,  8-10 wks | 5×10^7^ vp/mouse  100 nl, ic | 7-14 d | Y (IHC) | NR | - | NR | - | (Li et al., 2023) |
| **CAG**-eGFP, **RSV**-shIcosl, non-GFP control (Viral Vector Production Unit, Universitat Autònoma Barcelona, Spain) | A20^ΔCNS-EC^ mice, adult? | 1.8×10^11^gp  100μl, tail vein | 4 wks | Y (IHC, BC) | N | - | NR | - | (Johann et al., 2023) |
| CAG-eGFP (SignaGen, MD) | C57BL/6 mice, 6-8 wk | 0.1-1×10^12^ vg/mouse, 100μl retro-orbital | 3 wks | Y (IHC) | Y | Neurons, astrocytes (IHC) | NR | - | (Chen et al., 2023) |
| **CAG**-GFP (Dr. Korbelin) | C57BL/6 mice, P30 | 3×10^12^ gc/ml 100μl, retro-orbital | 4 wks | Y (IHC) | Y | Retinal ganglion cells (IHC) | NR | - | (Toma et al., 2024) |
|  |  | 3×10^12^ gc/ml 100μl intra-ocular | 4 wks | N (IHC) | Y | Retinal neurons (IHC) | NR | - |  |
| **CAG**-GFP (SignaGen, MD) | Sprague Dawley rats, 25 d | 1.5×10^11^, 3×10^11^, 4.5x10^11^ vg/mouse, tail vein | 3 wks | N (IHC) | Y | Neurons (IHC) | NR | - | (Kremer and Williams, 2024) |

**Key**: *Minimal, **AAV**, Adeno-associated virus; **CAG**, chicken beta actin promoter; **CMV**, cytomegalovirus promoter; **d,** day; **gc**, genome copies; **GFP**, Green fluorescent protein**; GP**, genome particles**; IC**, intracerebral; **ICV**, intracerebroventricular; **IHC**, immunohistochemistry; ISH, in situ hybridization; **KO**, knock-out; **m**, month; **NR**, not reported; **vg**, viral genome; **wk**, week

**Table S2. Summary of published data for AAV-BI30 selectivity in rodents**

| **AAV construct** | **Model, Age at Tx** | **Dose, route of admin** | **AAV duration** | **BEC expression** | **Non-BEC expression (CNS)** | | **Peripheral expression** | | **Ref** |
| --- | --- | --- | --- | --- | --- | --- | --- | --- | --- |
|  |  |  |  |  | **Y/N/NR** | **Cell Type** | **Y/N/NR** | **Organ** |  |
| **CAG**-NLS-GFP | C57Bl/6 mice, adult | 1x10^11^vg/mouse, tail vein | 10 d | Y (IHC) | NR | - | Y | Liver (IHC) | (Krolak et al., 2022) |
| **CAG**-NLS-GFP-miR122-WPRE | BALB/cJ mice, adult |  | 10 d | Y (IHC) | NR | - | NR | - |  |
|  | C57Bl/6 mice, adult |  | 3 wks | Y (IHC) | N | - | N | Liver (IHC) |  |
|  | Cav1^fl/fl^ | 1x10^11^vg/mouse, tail vein | 4 wks | Y (IHC) | N | - | NR | - |  |
| BI30, source unknown | C57Bl/6 mice | 1x10^12^ vg/animal , tail vein, 30-40 d | 2 wks | Y (IHC) | NR | - | Y | Liver (IHC) | (Giannelli et al., 2024) |

**Key**: *Minimal, **AAV**, Adeno-associated virus; **CAG**, chicken beta actin promoter; **CMV**, cytomegalovirus promoter; **d,** day; **gc**, genome copies; **GFP**, Green fluorescent protein**; GP**, genome particles**; IC**, intracerebral; **ICV**, intracerebroventricular; **IHC**, immunohistochemistry; **KO**, knock-out; **m**, month; **NR**, not reported; **vg**, viral genome; **wk**, week


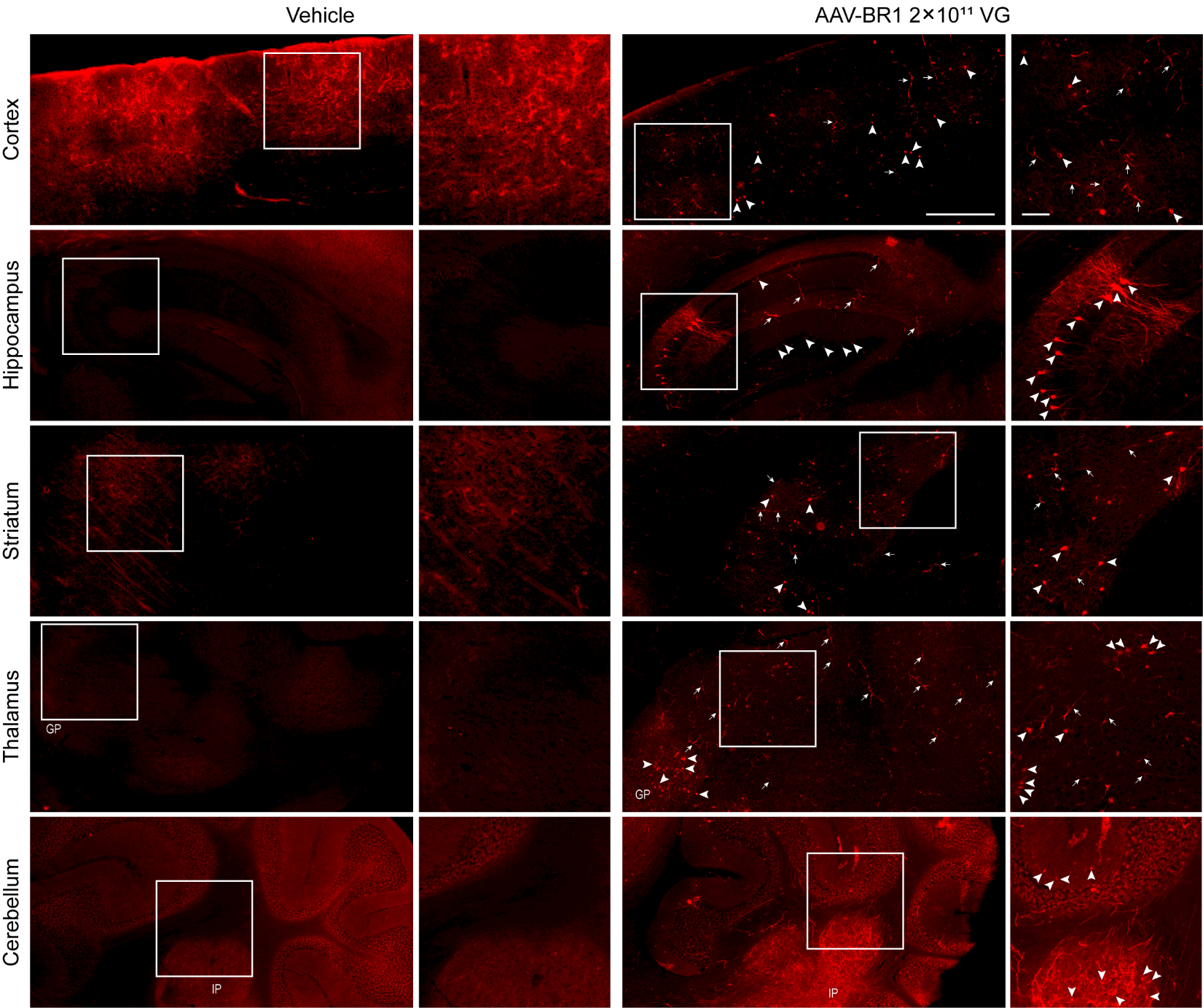


**Supplementary Figure 1. AAV-BR1 demonstrates brain endothelial and neuronal tropism in multiple brain regions.** AAV-BR1-mCherry has neuronal tropism (arrowheads) in multiple brain regions alongside brain endothelial cell tropism (arrows) without the need for further immunohistochemical staining for RFP. Images acquired in 594 channel. Scale bar = 500 µm and 100 µm (zoom-in). GP = globus pallidus; IP = interposed nucleus. Note, signal in the vehicle is autofluorescence.


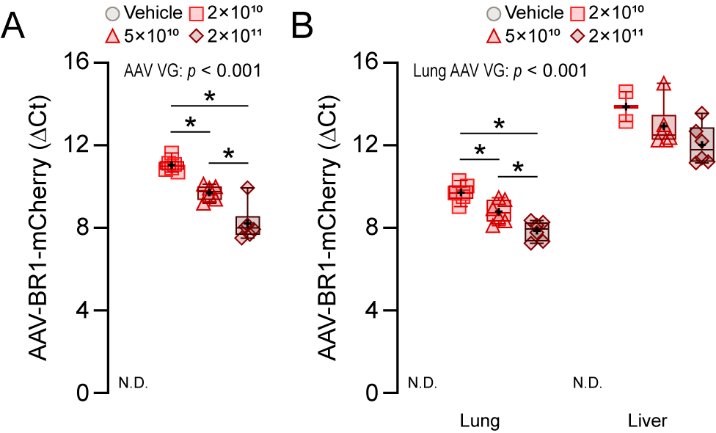


**Supplementary Figure 2. AAV-BR1 doses with vg load. A**, AAV-BR1-mCherry expression levels in the cortex measured by RT-PCR. ΔCt values decrease with increasing AAV-BR1-mCherry vg in a dose-dependent manner in the cortex [*F*_(2,17)_ = 35.865; *p* < 0.001], *n* = 6. AAV-BR1-mCherry is not detected in vehicle-treated mice in the cortex. **B**, AAV-BR1-mCherry expression levels in the lung and liver measured by RT-PCR. ΔCt values decrease with increasing AAV-BR1-mCherry vg in a dose-dependent manner in the lung [*F*_(2,17)_ = 21.241; *p* < 0.001], *n* = 6, and there is a slightly trending decrease in the liver [*F*_(2,13)_ = 2.796; *p* = 0.104], *n* ≈ 6. AAV-BR1-mCherry is not detected in vehicle-treated mice in the lung or liver. Data are expressed as a box plot depicting the minimum score, the lower quartile (25%), the median (50%, horizontal line), the upper quartile (75%), maximum values, and mean (+). Data were analyzed using GLM.


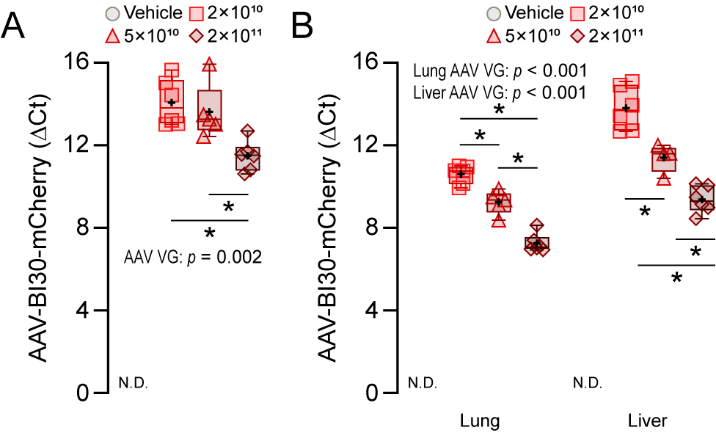


**Supplementary Figure 3. AAV-BI30 doses with vg load. A**, AAV-BI30-mCherry expression levels in the cortex measured by RT-PCR. ΔCt values decrease with increasing AAV-BI30-mCherry vg in a dose-dependent manner in the cortex [*F*_(2,16)_ = 9.645; *p* = 0.002], *n* ≈ 6. AAV-BI30-mCherry is not detected in vehicle-treated mice in the cortex. **B**, AAV-BI30-mCherry expression levels in the lung and liver measured by RT-PCR. ΔCt values decrease with increasing AAV-BI30-mCherry vg in a dose-dependent manner in the lung [*F*_(2,16)_ = 72.19; *p* < 0.001], *n* = 6, and liver [*F*_(2,15)_ = 41.788; *p* <0.001], *n* ≈ 6. AAV-BI30-mCherry is not detected in vehicle-treated mice in the lung or liver. Data are expressed as a box plot depicting the minimum score, the lower quartile (25%), the median (50%, horizontal line), the upper quartile (75%), maximum values, and mean (+). Data were analyzed using GLM.


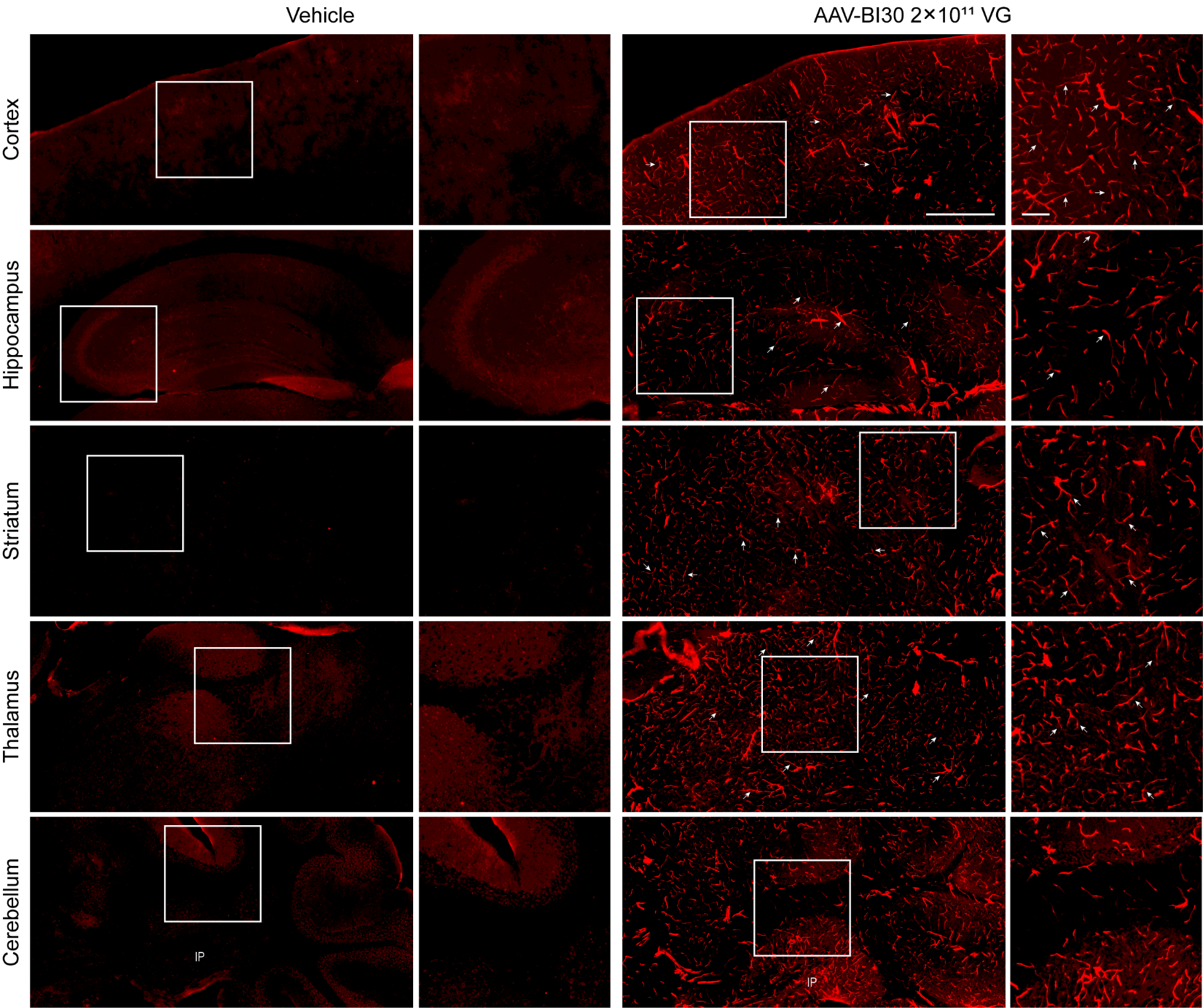


**Supplementary Figure 4. AAV-BI30 tropism within the brain is endothelial cell-specific.** AAV-BI30-mCherry shows specific tropism for brain endothelial cells (arrows) in tissue sections labeled for RFP by immunohistochemical staining. Scale bar = 500 µm and 100 µm (zoom-in). GP = globus pallidus. Images acquired in 647 channel.

**Major Resources Table**

**Animals (in vivo studies)**

| **Species** | **Vendor or Source** | **Background Strain** | **Sex** | **Persistent ID / URL** |
| --- | --- | --- | --- | --- |
| Mice | Jackson Laboratory | C57BL/6J | female | https://www.jax.org/strain/000664 |

**Genetically Modified Animals: N/A**

|  | **Species** | **Vendor or Source** | **Background Strain** | **Other Information** | **Persistent ID / URL** |
| --- | --- | --- | --- | --- | --- |
| **Parent - Male** |  |  |  |  |  |
| **Parent - Female** |  |  |  |  |  |

**Antibodies**

| **Target antigen** | **Application** | **Vendor or Source** | **Catalog #** | **Working concentration** | **Lot # (preferred but not required)** | **Persistent ID / URL** |
| --- | --- | --- | --- | --- | --- | --- |
| CD31 | IHC | R&D Systems | AF3628 | 1:250 |  | <https://www.rndsystems.com/products/human-mouse-rat-cd31-pecam-1-antibody_af3628>  RRID:AB2161028 |
| RFP | IHC | Proteintech | 5F8 | 1:250 |  | <https://www.ptglab.com/products/RFP-antibody-5F8.htm>  RRID:AB_2336064 |

**Gene Expression Assays**

| **Assay ID** | **Vendor or Source** | **Gene ID** | **Gene Name** | **Encoded Protein** |
| --- | --- | --- | --- | --- |
| Mm02619580_g1 | TaqMan, Thermo Fisher Scientific | ENSMUSG00000029580 | *Actb* | β-actin |
| Mr07319439_mr | TaqMan, Thermo Fisher Scientific |  | *Mcherry* | Cherry Fam |

**DNA/cDNA Clones : N/A**

**Cultured Cells: N/A**

**Data & Code Availability: N/A**

**Other: N/A**

| **Description** | **Source / Repository** | **Persistent ID / URL** |
| --- | --- | --- |
| AAV-(BR1)-CMV-P2A-mCherry | SignaGen  SL116135 | https://signagen.com/Pre-made-AAVs/AAV-CMV-mCherry-AAV-Serotype-BR1 |
| AAV-(BI30)-CMV-P2A-mCherry-3xmiR122 | SignaGen | None |

**ARRIVE GUIDELINES**

**Study Design**

Our study design is detailed in the methods and all details on n size, statistics and outliers that were removed are presented in the Source Data File 1. All mice in the study were cage mates. Our mice were purchased from The Jackson Laboratory at 3 weeks of age and housed in cages of 4-5 mice. When the mice reached 6 weeks of age they were rehoused in cages of 3 mice and treated with AAV by retro-orbital injection. Experiments were carried out at 10 weeks of age.

| **Groups** | **Sex** | **Age** | **Number (prior to experiment)** | **Number (after termination)** | **Littermates**  **(Yes/No)** | **Other description** |
| --- | --- | --- | --- | --- | --- | --- |
| C57BL/6J (Control) | Female | 10 weeks | 36 | 65 | Cage mates. Mice were purchased so littermate status is not confirmed. |  |

**Sample Size:**

n = 6 per AAV per dose

**Inclusion Criteria**

N/A

**Exclusion Criteria**

Mice that were separated due to fighting or died during the study were excluded.

**Randomization**

Cages contained 4-5 mice at the start of housing prior to treatment with AAV. Mice were then rehoused into cages of 3 mice. Each cage was randomly assigned an AAV and dose.

**Blinding**

For quantification of all experiments, investigators were blinded to AAV and dose.

**References**

Alvarez-Vergara MI et al. (2021) Non-productive angiogenesis disassembles Ass plaque-associated blood vessels. Nat Commun 12:3098.

Chen DY, Sun NH, Chen X, Gong JJ, Yuan ST, Hu ZZ, Lu NN, Korbelin J, Fukunaga K, Liu QH, Lu YM, Han F (2021) Endothelium-derived semaphorin 3G attenuates ischemic retinopathy by coordinating beta-catenin-dependent vascular remodeling. J Clin Invest 131.

Chen X, He Y, Tian Y, Wang Y, Wu Z, Lan T, Wang H, Cheng K, Xie P (2020) Different Serotypes of Adeno-Associated Virus Vector- and Lentivirus-Mediated Tropism in Choroid Plexus by Intracerebroventricular Delivery. Hum Gene Ther 31:440-447.

Chen X et al. (2023) Functional gene delivery to and across brain vasculature of systemic AAVs with endothelial-specific tropism in rodents and broad tropism in primates. Nat Commun 14:3345.

Dogbevia G, Grasshoff H, Othman A, Penno A, Schwaninger M (2020) Brain endothelial specific gene therapy improves experimental Sandhoff disease. J Cereb Blood Flow Metab 40:1338-1350.

Dogbevia GK, Tollner K, Korbelin J, Broer S, Ridder DA, Grasshoff H, Brandt C, Wenzel J, Straub BK, Trepel M, Loscher W, Schwaninger M (2017) Gene therapy decreases seizures in a model of Incontinentia pigmenti. Ann Neurol 82:93-104.

Giannelli SG, Luoni M, Iannielli A, Middeldorp J, Philippens I, Bido S, Korbelin J, Broccoli V (2024) New AAV9 engineered variants with enhanced neurotropism and reduced liver off-targeting in mice and marmosets. iScience 27:109777.

Huo R, Yang Y, Sun Y, Zhou Q, Zhao S, Mo Z, Xu H, Wang J, Weng J, Jiao Y, Zhang J, He Q, Wang S, Zhao J, Wang J, Cao Y (2023) Endothelial hyperactivation of mutant MAP3K3 induces cerebral cavernous malformation enhanced by PIK3CA GOF mutation. Angiogenesis 26:295-312.

Ivanova E, Corona C, Eleftheriou CG, Stout RF, Jr., Korbelin J, Sagdullaev BT (2022) AAV-BR1 targets endothelial cells in the retina to reveal their morphological diversity and to deliver Cx43. J Comp Neurol 530:1302-1317.

Johann L et al. (2023) A20 regulates lymphocyte adhesion in murine neuroinflammation by restricting endothelial ICOSL expression in the CNS. J Clin Invest 133.

Kawabata H, Konno A, Matsuzaki Y, Hirai H (2023) A blood-brain barrier-penetrating AAV2 mutant created by a brain microvasculature endothelial cell-targeted AAV2 variant. Mol Ther Methods Clin Dev 29:81-92.

Korbelin J, Dogbevia G, Michelfelder S, Ridder DA, Hunger A, Wenzel J, Seismann H, Lampe M, Bannach J, Pasparakis M, Kleinschmidt JA, Schwaninger M, Trepel M (2016) A brain microvasculature endothelial cell-specific viral vector with the potential to treat neurovascular and neurological diseases. EMBO Mol Med 8:609-625.

Kremer R, Williams A (2024) AAV-BR1 does not target endothelial cells in Sprague Dawley rats unlike in mice. MicroPubl Biol 2024.

Krolak T, Chan KY, Kaplan L, Huang Q, Wu J, Zheng Q, Kozareva V, Beddow T, Tobey IG, Pacouret S, Chen AT, Chan YA, Ryvkin D, Gu C, Deverman BE (2022) A High-Efficiency AAV for Endothelial Cell Transduction Throughout the Central Nervous System. Nat Cardiovasc Res 1:389-400.

Li L, Ren AA, Gao S, Su YS, Yang J, Bockman J, Mericko-Ishizuka P, Griffin J, Shenkar R, Alcazar R, Moore T, Lightle R, DeBiasse D, Awad IA, Marchuk DA, Kahn ML, Burkhardt JK (2023) mTORC1 Inhibitor Rapamycin Inhibits Growth of Cerebral Cavernous Malformation in Adult Mice. Stroke 54:2906-2917.

Liu XX, Yang L, Shao LX, He Y, Wu G, Bao YH, Lu NN, Gong DM, Lu YP, Cui TT, Sun NH, Chen DY, Shi WX, Fukunaga K, Chen HS, Chen Z, Han F, Lu YM (2020) Endothelial Cdk5 deficit leads to the development of spontaneous epilepsy through CXCL1/CXCR2-mediated reactive astrogliosis. J Exp Med 217.

Mehina EMF, Taylor S, Boghozian R, White E, Choi SE, Cheema MS, Korbelin J, Brown CE (2021) Invasion of phagocytic Galectin 3 expressing macrophages in the diabetic brain disrupts vascular repair. Sci Adv 7.

Nikolakopoulou AM, Wang Y, Ma Q, Sagare AP, Montagne A, Huuskonen MT, Rege SV, Kisler K, Dai Z, Korbelin J, Herz J, Zhao Z, Zlokovic BV (2021) Endothelial LRP1 protects against neurodegeneration by blocking cyclophilin A. J Exp Med 218.

Park ES, Kim S, Huang S, Yoo JY, Korbelin J, Lee TJ, Kaur B, Dash PK, Chen PR, Kim E (2021) Selective Endothelial Hyperactivation of Oncogenic KRAS Induces Brain Arteriovenous Malformations in Mice. Ann Neurol 89:926-941.

Rasmussen CLM, Hede E, Routhe LJ, Korbelin J, Helgudottir SS, Thomsen LB, Schwaninger M, Burkhart A, Moos T (2023) A novel strategy for delivering Niemann-Pick type C2 proteins across the blood-brain barrier using the brain endothelial-specific AAV-BR1 virus. J Neurochem 164:6-28.

Ren AA et al. (2021) PIK3CA and CCM mutations fuel cavernomas through a cancer-like mechanism. Nature 594:271-276.

Santisteban MM, Ahn SJ, Lane D, Faraco G, Garcia-Bonilla L, Racchumi G, Poon C, Schaeffer S, Segarra SG, Korbelin J, Anrather J, Iadecola C (2020) Endothelium-Macrophage Crosstalk Mediates Blood-Brain Barrier Dysfunction in Hypertension. Hypertension 76:795-807.

Song X, Cui Y, Wang Y, Zhang Y, He Q, Yu Z, Xu C, Ning H, Han Y, Cai Y, Cheng X, Wang J, Teng Y, Yang X, Wang J (2022) Genome Editing with AAV-BR1-CRISPR in Postnatal Mouse Brain Endothelial Cells. Int J Biol Sci 18:652-660.

Sundaram SM, Arrulo Pereira A, Muller-Fielitz H, Kopke H, De Angelis M, Muller TD, Heuer H, Korbelin J, Krohn M, Mittag J, Nogueiras R, Prevot V, Schwaninger M (2022) Gene therapy targeting the blood-brain barrier improves neurological symptoms in a model of genetic MCT8 deficiency. Brain 145:4264-4274.

Tan C, Lu NN, Wang CK, Chen DY, Sun NH, Lyu H, Korbelin J, Shi WX, Fukunaga K, Lu YM, Han F (2019) Endothelium-Derived Semaphorin 3G Regulates Hippocampal Synaptic Structure and Plasticity via Neuropilin-2/PlexinA4. Neuron 101:920-937 e913.

Toma K, Zhao M, Zhang S, Wang F, Graham HK, Zou J, Modgil S, Shang WH, Tsai NY, Cai Z, Liu L, Hong G, Kriegstein AR, Hu Y, Korbelin J, Zhang R, Liao YJ, Kim TN, Ye X, Duan X (2024) Perivascular neurons instruct 3D vascular lattice formation via neurovascular contact. Cell 187:2767-2784 e2723.

Wang J et al. (2019) Brain Endothelial Cells Maintain Lactate Homeostasis and Control Adult Hippocampal Neurogenesis. Cell Stem Cell 25:754-767 e759.
